# Micro-Fragmented Adipose Tissue (MFAT) in Orthopedic Regenerative Medicine: A Narrative Review of the Biological Basis and Clinical Evidence

**DOI:** 10.3390/ijms27146185

**Published:** 2026-07-10

**Authors:** Claire Yuan, Ashu K. Goyle, Maged Guirguis, Alan D. Kaye, Vahid Grami, Karan Dave, Ronald J. Kulich, Timothy Deer, David Rosenblum, Vwaire Orhurhu, Jamal J. Hasoon, Christopher L. Robinson

**Affiliations:** 1 Department of History and Philosophy of Science, Christ’s College, University of Cambridge, Cambridge CB2 3RH, UK; 2 Integrated Spine, Pain and Wellness, Scottsdale, AZ 85260, USA; 3 Department of Interventional Pain, Oschner Health System, New Orleans, LA 70121, USA; 4 Department of Anesthesiology, Louisiana State University Health Sciences Center Shreveport, Shreveport, LA 71103, USA; 5 Division of Pain Medicine, Department of Anesthesiology and Critical Care, The Johns Hopkins University School of Medicine, Baltimore, MD 21287, USA; 6 Aurora Life, Grand Cayman KY1-1209, Cayman Islands; 7 Department of Anesthesia, Critical Care, and Pain Medicine, Massachusetts General Hospital, Boston, MA 02114, USA; 8 Department of Psychiatry, Massachusetts General Hospital, Boston, MA 02114, USA; 9 Spine and Nerve Center of the Virginias, West Virginia University—Health Sciences Campus, Morgantown, WV 25301, USA; 10 Department of Anesthesiology, Maimonides Medical Center, Brooklyn, NY 11219, USA; 11 MVM Health: Pain Vein, and Wellness, East Stroudsburg, PA 18301, USA; 12 Department of Anesthesia, Critical Care, and Pain Medicine, UTHealth, McGovern Medical School, Houston, TX 77030, USA

**Keywords:** micro-fragmented adipose tissue, regenerative medicine, osteoarthritis, mesenchymal stem cells

## Abstract

Micro-fragmented adipose tissue (mFAT) is a promising autologous biologic in regenerative medicine because it provides a mechanically processed adipose-derived product that preserves native extracellular matrix architecture and a cellular milieu rich in mesenchymal stem cells, pericytes, growth factors, cytokines, and extracellular vesicles. Mechanistically, mFAT is hypothesized to act largely through paracrine signaling that dampens inflammation, supports vascular stabilization, and promotes cartilage and soft-tissue repair; in vitro data suggest modulation of osteoarthritic synovial macrophage signaling, including reductions in chemokines such as CCL2 and CCL3. Preparation involves liposuction harvest followed by closed, sterile mechanical processing without enzymatic digestion or cell expansion, aligning with “minimal manipulation” concepts relevant to regulatory frameworks. Preclinical animal studies generally demonstrate favorable effects on synovial inflammation and cartilage matrix markers (e.g., glycosaminoglycan content) with limited adverse events. Clinically, the strongest body of evidence is in knee osteoarthritis, where multiple prospective and retrospective studies report improvements in pain and function from months to several years after single injections, though response rates vary and study designs are heterogeneous. Evolving data support potential benefit in hip osteoarthritis and select tendon conditions, but cohorts remain small. Overall, mFAT appears safe and potentially effective, yet larger, standardized, long-term randomized controlled trials and comparative studies versus platelet-rich plasma and bone marrow aspirate concentrates are needed to clarify indications, dosing, durability, and mechanisms in vivo.

## 1. Introduction

Mesenchymal stem cells (MSCs) play a vital role in tissue repair, particularly as it relates to bone, cartilage, heart, and vascular systems by differentiating into various cell types including myoblasts, adipocytes, chondrocytes, and osteoblasts [1]. Moreover, MSCs can secrete several biologically active molecules that have the capacity to modulate the immune system and effect tissue healing and regeneration [1]. Many tissues of the body can be processed to yield MSCs, but in the case of mFAT, they are specifically derived from adipose tissue [1]. As a result of its accessibility and relatively high cell yield, adipose tissue is a good candidate. Indeed, studies indicate that one gram of adipose tissue yields several thousand stem cells [1].

There are multiple adipose-derived biologics that have been studied in regenerative medicine for better wound healing, including but not limited to micro-fragmented adipose tissue (mFAT) and stromal vascular fraction (SVF). The present investigation focuses on mFAT, which retains the structural collagens and microenvironment of the adipose tissue and appears to provide benefits for healing over alternatives such as SVF [2]. It is also of note that mFAT is an autologous fat derivative, therefore avoiding rejection reactions that can arise when biologics are produced from foreign sources [3].

In addition to the MSCs derived from adipose, mFAT is also rich in pericytes, growth factors, extracellular vesicles (including exosomes, microvesicles, etc.), and a complete and intact extracellular matrix for cell proliferation in healing [3]. The increased number of cytokines and extracellular vesicles in mFAT compared to standard enzymatic treatments can result in a heightened capacity for tissue repair and regeneration, including angiogenesis, stem cell transformation, and homing [4]. The abundance of pericytes, as demonstrated in in vitro studies, indicated the potential for further differentiation into chondrogenic, osteogenic, and adipogenic lineages [5,6,7].

Studies have indicated that mFAT has strong anti-inflammatory and anti-apoptotic properties, as well as the ability to release extracellular vesicles, cytokines, and various other regulatory factors to promote tissue and cartilage regeneration in the local environment of the injury site [8,9,10,11,12]. Additionally, mFAT has been shown to release mediators with longer-lasting anti-inflammatory properties than MSCs alone when placed under serum-free cell culture conditions, thereby further promoting the potential for mFAT [13].

Though there is significant further study needed, mFAT has been shown in preclinical and clinical studies to have potential in the treatment of joint-, cartilage-, and tendon-related injuries and conditions. In this regard, mFAT shows potential for being less invasive than traditional therapies for conditions such as osteoarthritis and total knee arthroplasty [14,15]. Intra-articular injections of hyaluronic acid or corticosteroids typically are only capable of providing alleviation and benefits in the short term, and use of non-steroidal anti-inflammatory drugs (NSAIDs) can result in side effects with chronic use (and are not capable of stopping tissue degeneration) [16,17,18].

Nonetheless, like many other new biologic therapies in regenerative and pain management medicine, mFAT similarly requires further research to better characterize its effectiveness across a range of conditions and over a longer course of time. As part of this, investigators must pursue larger and more encompassing randomized controlled trials.

## 2. Methods

This narrative review was conducted through searches of multiple databases, including PubMed, the Cochrane Library, and Google Scholar through March 2026. Search terms included: “micro-fragmented adipose tissue,” “mFAT,” “micro-fragmented fat,” “adipose-derived stem cells,” and “stromal vascular fraction.” English-language peer-reviewed publications were prioritized in consideration. These included randomized controlled trials, prospective and retrospective studies, systematic reviews, meta-analyses, and relevant in vitro and preclinical studies. In identified narrative and systematic reviews, reference lists were searched thoroughly for additional relevant sources. No formal PRISMA-based screening process, risk-of-bias assessment, or meta-analytic pooling was performed; the review aims to synthesize the available evidence narratively.

### 2.1. Preparation and Administration

**Figure 1:**
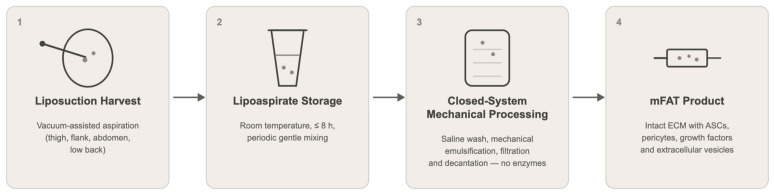
Stages of micro-fragmented adipose tissue preparation.

Among the main advantages of mFAT are that (1) it is autologous and avoids the risk of rejection and (2) it is comparably much simpler to prepare. To generate a sample of autologous mFAT for a patient, the adipose tissue must first be harvested via a small-scale liposuction (Figure 1). This can be done using vacuum aspiration with a cannula, typically from areas of the body such as the inner and outer thighs, flanks, abdomen, and mid and lower back [2]. Harvest site complications may occur, including infection, hematoma, and contour abnormalities if done too superficially [19]. Typical processing yields between 50 and 120 mL of lipoaspirate harvested, which can produce about 5 to 20 mL of mFAT product [19,20]. Prior to further processing, lipoaspirate can be stored at room temperature for up to 8 h, with thorough mixing to ensure that homogeneity of the sample is maintained [2].

**Figure 2:**
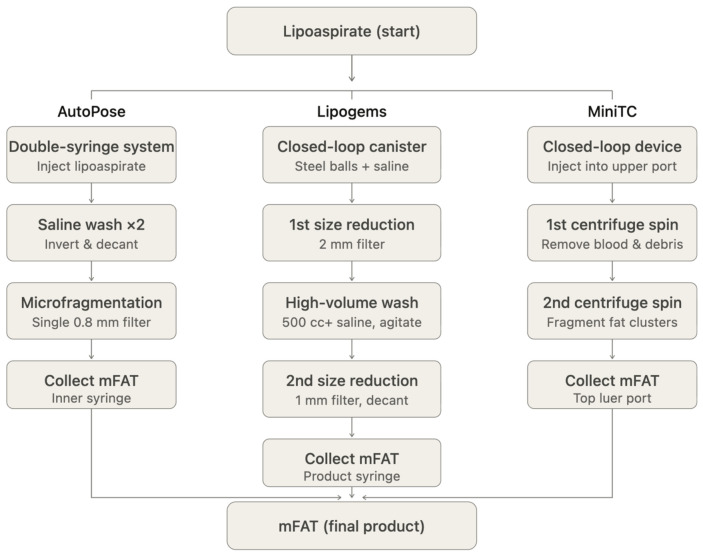
Comparison of mFAT processing pathways: AutoPose, Lipogems, and MiniTC.

Production of mFAT from the lipoaspirate involves mechanical processing, for which LipoGems^®^, AutoPose™, and MiniTC^®^ processing technologies are often used (Figure 2 and Table 1). In AutoPose™, the lipoaspirate is injected into the system and washed by the subsequent injection of sterile saline and allowing for gravimetrical separation. Then, the intermediate is decanted and filtered to obtain the final mFAT product [2]. The LipoGems^®^ system is now particularly popular due to its use of a sterile, closed environment to mechanically process the lipoaspirate. In this closed-loop system, sterile saline is used to rinse the adipose sample, which is shaken in a canister. This process is repeated several times and decanted to obtain the final mFAT product [2]. In either case, there is very limited processing necessary. For MiniTC^®^, the process is similar, as it is a closed-loop system and involves centrifuging, washing, removing debris, and isolating the mFAT for injection all within 30 min [21]. Importantly, mFAT simply needs to be treated with mild mechanical force and does not require any more complex procedures, such as enzyme treatment or cell expansion [3,22]. This is a promising advantage of this biologic, as the processing of mFAT removes blood elements but retains tissue structure and stromal cell populations [2,23].

Notably, Ragni et al. found 376 and 381 miRNAs, contained in extracellular vesicles in mFAT, which were detected in lipoaspirate and mFAT samples [23]. To characterize the mFAT product produced, studies have often relied on techniques like flow cytometry and cell culture [2]. However, across various processing devices and methodologies, cellular composition and cytokine secretion profiles can be distinct, leading to variations that lack standardized analyses and present a major challenge for direct comparisons in the field [2].

To avoid the need for repeated liposuction when repeated injections are necessary for treatment, mFAT samples obtained autologously can be stored at −80 °C in a tissue bank with no significant alteration in viability, though the average cell count is lower in thawed samples compared to fresh ones [24]. Further characterization remains necessary to assess the exact differences in efficacy, if any, as a result of freezing and thawing. Once prepared, the mFAT can then be administered to the patient, typically via intra-articular—and in some cases, peri-articular—injections to the affected tissues and joints. While there is not a standardized consensus on dosage and concentration, the most reported volumes injected range from 5 to 9 mL per injection for a total volume of 15 to 20 mL [19]. At the administration site, potential adverse effects include infection and joint effusion, though most preclinical and clinical studies have not found any significant complications, as will be narratively reviewed subsequently in the paper [19].

Despite the comparative ease of preparing mFAT as a regenerative biologic, there nonetheless remain concerns about standardization for more widespread use and application. Variations in harvesting technique, harvesting location, processing methods (e.g., enzymatic digestion, mechanical emulsification, centrifugation, and washing), and differences in donor biology may result in inconsistent mFAT production [2,25]. For instance, some researchers have noted that body mass index may be important for the preparation and administration of mFAT. BMI is known to contribute to patient outcomes for knee osteoarthritis (a significant area of study and promise for mFAT use), as well as the protein expression of orthobiologic treatments such as mFAT, thereby raising questions around its effects on efficacy [26]. Baria et al. [26] conducted a secondary analysis including 71 patients with knee osteoarthritis to receive an injection of either PRP or mFAT, after which patient reports and Knee injury and Osteoarthritis Outcome Scores (KOOSs) were tracked for one year. Of the 49 patients who completed the 12-month follow-up, researchers found KOOS and quality of life were inversely correlated with BMI for mFAT-treated patients but not PRP-treated patients [26].

**Table 1:** Overview of commercially available mFAT processing systems.

Device	Key Features	Processing Time	Cost	Advantages
LipoGems^®^ [2,5]	Closed-loop saline washing and mechanical fragmentation	~15–20 min	~$1500–$2500	Sterile system; widely used; preserves microarchitecture
AutoPose^TM^ [2,27]	Saline wash, decanting, filtration	~10–20 min	~$1200–$2000	Simple workflow; efficient separation
MiniTC^®^ [5,21]	Closed system with centrifugation, washing, debris removal (~30 min)	~5–10 min	~$500–$1000	Rapid processing; integrated system

#### 2.1.1. Mechanisms of Action

To understand mFAT’s effects in the body, it is essential to characterize its cell population contents. According to guidelines from the International Federation for Adipose Therapeutics and Science (IFATS) and the International Society for Cellular Therapy (ISCT), adipose-derived mesenchymal stromal cells in mFAT are identified by positive surface expression of CD105, CD90, and CD73, as well as negative expression of CD45 and CD34 [28]. Further, pericytes in mFAT are identified by the expression of CD146 and NG2 [23]. Using flow cytometry, Ragni et al. confirmed that both ASCs and pericytes are maintained in mFAT at proportions comparable to those present in unprocessed lipoaspirate, suggesting that mechanical fragmentation does not deplete or enrich specific subpopulations [23]. It remains important to note that ASCs isolated from mFAT retain their capacity for differentiation (osteogenic, adipogenic, and chondrogenic), which is crucial for function [23].

While the mechanisms of action for mFAT are not well characterized, some investigators have begun to hypothesize the paracrine role of the biologic. In an in vitro study, Bosetti et al. studied the potential of mFAT by culturing clusters of lipoaspirate, which showed a spontaneous outgrowth of mesenchymal phenotypic cells with the ability to differentiate into a variety of lineages [29]. The researchers suggest that the lipoaspirate may have a paracrine effect.

Similarly, Ceserani et al. [30] suggested that mFAT acts through paracrine action to induce vascular stabilization and prevent inflammation [30]. In particular, the active stromal cells like adipose-derived stem cells (ASCs) and pericytes provide paracrine action, as ASCs can differentiate into a wide range of cell types—including neuronal cells, cardiomyocytes, epithelial cell types, and more—as well as demonstrating immunomodulatory and anti-inflammatory effects [1,23].

Compared to lipoaspirate, mFAT was seen by Nava et al. to produce significantly greater levels of G-CSF, SCGF-β, and HGF, stimulating the production and activation of mesenchymal stem cells, increasing stem cell growth factor expression, and boosting tissue recruitment [13].

Further, over time, the cultured lipoaspirate developed a different structure, increasing the amount of connective tissue rich in glycosaminoglycan (GAG) and collagen, indicating an improvement in mechanical strength [29]. These findings, the investigators conclude, demonstrate potential indications for the ability of mFAT to become a fibrous tissue to support damaged cartilage during healing, cause host chondrocytes to proliferate and generate new extracellular matrix, and provide cells to regenerate and repair at the injury site [29].

In the context of osteoarthritis, pro-inflammatory M1 macrophages release TNF-α, IL-1β, and IL-6 to perpetuate cartilage degradation and synovitis. In contrast, M2 macrophages secrete anti-inflammatory mediators such as IL-10 and TGF-β to promote tissue repair and resolution of inflammation [31]. Adipose-derived MSC secretome components, particularly TSG-6, TGF-β, and PGE2, have been shown to drive a phenotypic switch from M1 to M2 macrophages [32]. Specifically, TSG-6 released from adipose-derived MSCs induces M2 polarization, characterized by upregulation of mannose receptor CD20 and elevated IL-10 expression, while suppressing TNF-α and IL-1β [7,32].

Moreover, in an in vitro study of osteoarthritic synovial cells, Paolella et al. reported that mFAT plays an active role in mediating synovial macrophage activity due to a decrease in CCL2/MCP1 and CCL3/MIP1α [7]. These chemokines are M1-associated, and their suppression therefore represents a dampening of M1-driven synovial inflammation, consistent with macrophage polarization of M2 [7]. Guo et al. also note that in LPS-stimulated macrophage models, mFAT-conditioned medium attenuates the release of pro-inflammatory cytokines [31].

#### 2.1.2. Preclinical Characterizations in Animal Models

Multiple studies have been conducted on rabbits with various orthopedic defects. Desando et al. evaluated the efficacy of injecting expanded-adipose stromal cells, SVF, and mFAT in bilateral anterior cruciate ligament transection-induced osteoarthritis in adult New Zealand rabbits [33] (Table 2). All three biologics were found to have good cell viability and progenitor marker expression [33]. However, mFAT and SVF showed distinctive cell migration patterns at 7- and 30-day time points after the intra-articular injection of the different treatments into the knee joint, suggesting the need for further investigation to understand the underlying mechanisms for such differences in biology [33]. In particular, at 7 days, ASCs and SVF migrated mainly to the inflamed synovium, while at 30 days, mFAT showed more cartilage-associated localization. Nonetheless, mFAT, alongside the others, seemed to present the appropriate repair responses in osteoarthritic joints [33]. Filardo et al. similarly studied mFAT in rabbit models (96 knees) and found that mFAT can diminish synovial inflammation in rabbits, as well as playing a part in protecting cartilage [34]. In particular, two years after one singular intra-articular injection of mFAT, there were significantly increased levels of GAG in the articular cartilage [34]. This has positive implications for the ability of mFAT to support cartilage matrix synthesis and slow down the progression of osteoarthritis [34].

In addition, some researchers have investigated similar small animal models. Zeira et al. studied the use of a single intra- or peri-articular mFAT injection to treat spontaneous osteoarthritis in 130 dogs [35]. Clinical outcomes were followed for 6 months through orthopedic examination and owner testimony, both of which indicated that the vast majority of subjects showed considerable improvement compared to the baseline with no significant local or systemic complications [35]. In rats (n = 12), Xu et al. found that mFAT significantly boosted the migration of chondrocytes, as well as improving the visual appearance of damaged cartilage [36]. Cartilage defects were created on the femoral groove of rats and treated either with mFAT or PBS for 6 and 12 weeks [36]. Through evaluation by histological staining, immunohistochemistry, and scoring, researchers found that mFAT-treated subjects showed more regular cartilage surfaces and a large amount of hyaline cartilage, among other factors, that indicate the regeneration of normal, non-defective cartilage [36]. However, it must be noted that the sample size for this study is small, posing a significant limitation.

Overall, the limited preclinical data suggests that mFAT has a positive role in orthopedic healing with limited adverse effects, which has positive implications regarding supporting the findings in the clinical literature, as will be reviewed in the following section. However, it is also important to note that there is a significant base of preclinical literature that can be further built up for a better characterization of mFAT. In this regard, while in vitro studies have identified potential pathways and mechanisms of action for mFAT in wound healing, these mechanistic findings have been seen significantly less in live models in vivo.

**Table 2:** Summary of mFAT preclinical studies in animal models.

Study	Design	Intervention	Key Findings
Desando et al., 2019 [33]	Rabbits, bilateral ACL transection-induced osteoarthritis	Expanded-adipose stromal cells, SVF, mFAT	Observed at day 7, 30; all biologics showed good viability; mFAT contributed repair responses
Filardo et al., 2022 [34]	Rabbits (n = 96), synovial inflammation	Single mFAT intra-articular injection	Observed at 2, 4 months; reduced inflammation, protected cartilage; increased GAG levels (improved cartilage matrix synthesis)
Zeira et al., 2018 [35]	Dogs (n = 130), spontaneous osteoarthritis	Single intra- or peri-articular mFAT injection	Observed at 1, 6 months; significant clinical improvement; no major complications
Xu et al., 2019 [36]	Rats (n = 12), femoral groove cartilage defects	mFAT injection	Observed at 6, 12 weeks; improved cartilage structure; more hyaline cartilage; enhanced regeneration and repair

#### 2.1.3. Clinical Applications in the Previous Literature

Unlike some other biologics in regenerative medicine, mFAT has seen a reasonable amount of study in the clinical arena, though the studies tend to be small and short in follow-up time scale. Most of the focus around clinical applications of mFAT have circled around osteoarthritis (Table 3). The condition, which often results in significant pain and functional impairments in patients, has long been a focus due to its ability to significantly decrease quality of life [3,37]. As such, research has sought to continually produce improved methods of treatment and symptom alleviation. The pathological progression of osteoarthritis is characterized by the degradation of cartilage extracellular matrix (ECM), apoptosis of chondrocytes, the sole cell type that makes up articular cartilage, and release of inflammatory factors [3,38]. Since mFAT contains growth factors and an intact ECM structure, alongside numerous other regulatory molecules that can promote a positive microenvironment for injury healing, it is a promising alternative intervention for multiple types of osteoarthritis.

### 2.2. Knee Osteoarthritis

The use of mFAT has been most commonly studied for use in knee osteoarthritis, and there are a range of studies assessing the efficacy and safety of mFAT in the short and medium term for this application. De Groote et al. [14] conducted a longitudinal study on 39 patients with symptomatic Kellgren–Lawrence (KL) grade II-IV knee osteoarthritis who received single-dose mFAT injections. The KL system, graded from 0 to IV in increasing severity, is a radiographic grading scale for assessing knee osteoarthritis and focuses on structural damage [39]. Outcomes were assessed with KOOSs at baseline and 3, 6, and 12 months following treatment. Researchers reported that KOOSs improved across all subscales and remained high throughout the first year after mFAT application, though the highest scores occurred at 6 months. However, the study found that female patients showed worse outcomes than male patients. While significant adverse effects were not found, minor self-limiting synovitis lasting up to a maximum of 2 months occurred in 18% (n = 7) of patients [14].

Notably, the gender differences seen by De Groote et al. [14] are not consistent with other investigations of mFAT efficacy. Borg et al. reported that women rather responded more and with better outcomes to mFAT treatment for knee osteoarthritis [40]. The authors hypothesize that such gender differences lie in underlying genomic, hormonal, and metabolic factors, though they highlight the need for further research and characterization to truly understand these disparities [40].

Hudetz et al. conducted a prospective, non-randomized, interventional trial with 17 patients (for a total of 32 knees) with osteoarthritis [41]. Patients received intra-articular injection of mFAT and were assessed for VAS, with delayed gadolinium-enhanced magnetic resonance imaging of cartilage (dGEMRIC) and immunoglobulin G (IgG) glycans at baseline and 3, 6, and 12 months after administration of treatment [42]. The investigators suggested that the use of mFAT increased GAG content in hyaline cartilage. This is notable, as osteoarthritis is associated with the loss of macromolecules like GAG [42]. Heidari et al. similarly concluded that mFAT has potential in reducing the need for total knee replacements as a result of knee osteoarthritis [43].

Stanciu et al. reviewed mid-term efficacy, assessing the benefits of mFAT three years following application for knee osteoarthritis [44]. The retrospective, observational study included 335 patients who received one mFAT injection and were followed up with at 3 and 6 months and 1, 2, and 3 years [44]. Assessments were made using the Visual Analog Scale (VAS), Oxford Knee Score (OKS), Western Ontario and McMaster Universities Osteoarthritis Index (WOMAC), and KOOS [44]. The researchers found significant improvements throughout the three years, though a full statistical analysis was not possible due to study attrition over time [44].

Van Genechten et al. used autologous mFAT to investigate the short-term clinical effect, therapeutic response rate (TRR), and therapy safety on knee osteoarthritis [15]. Investigators used a single intra-articular autologous mFAT injection in a sample of 64 patients with symptomatic, mild-to-severe knee osteoarthritis (n =37 received a unilateral and n = 27 received a bilateral injection) [15]. Patients were clinically evaluated at 1, 3, 6, and 12 months after injection, at which time adverse effects and TRR were assessed [15]. Inflammation was reported in 79% of knees but resolved on its own within approximately two weeks of mFAT administration [15]. Researchers found that TRR was 64% at 3 months and 45% at 12 months post injection, which suggests that symptomatic improvement may decline over time, underscoring the need to identify predictors of durability and extended efficacy. Patients who responded to the therapy at 12 months improved with 28.3 ± 11.4 on the KOOS pain scale, while those who did not lost 2.1 ± 11.2 points [15]. Assessment of bone marrow lesions found a negative correlation with TRR at 12 months. Therefore, scientists suggest early clinical improvement from mFAT but mediocre response rate after one year [15]. As such, mFAT could present as a viable alternative for symptomatic knee osteoarthritis compared to repeat injections of cortisone, platelet-rich plasma (PRP), and hyaluronic acid.

Richter et al. performed a randomized controlled trial involving 75 patients with symptomatic knee osteoarthritis, grouped by baseline pain levels and split into mFAT, corticosteroid, and saline control injection treatment groups [45]. Using the visual analog pain scale, Western Ontario and McMaster Universities Osteoarthritis Index, and KOOS at 2 and 6 weeks and 3, 6, and 12 months, researchers found that mFAT resulted in statistically significant primary outcomes for both pain levels and joint functionality compared to the control group [45]. In comparison, the corticosteroid group showed only statistically significant improvement compared to the control at 2 and 6 weeks [45]. It is notable that Richter et al. [45] is one of few studies to have incorporated a true saline control. As such, the risk of inadequate study due to poor or non-existent controls and placebo effect is still salient. Future studies must consider and attempt to remedy this.

Some researchers have also focused on the use of mFAT in conjunction with existing methods of osteoarthritis treatment and symptom alleviation. A randomized controlled trial by Ulivi et al. reported that mFAT, administered alongside arthroscopic debridement, showed promise for the treatment of knee osteoarthritis [18]. The clinical study, which included 78 patients, was split into arthroscopic debridement only and arthroscopic debridement and mFAT testing groups [18]. At six months and 24 months following treatment, clinical, radiological, and serological assessments improved functional scores in the KOOS and Knee Society Score (KSS) measurements. Imaging from MRI scans also suggested visual improvement [18]. Hu et al. similarly found that use of mFAT injections combined with knee arthroscopy for knee osteoarthritis revealed significant pain relief and improved joint function with minimal complications in short-term follow-up [46]. Additionally, Cattaneo et al. studied 38 patients with symptomatic knee osteoarthritis who underwent an arthroscopic procedure associated with singular injection of mFAT [47]. Clinical outcomes, measured at 1, 3, 6, and 12 months afterwards, reported consistently improved KOOSs and no significant adverse effects and complications [47].

While most of the work in this arena has focused on short-term safety and efficacy, some investigators have also begun to investigate the mid- to long-term time frame. In a retrospective study of 49 patients including 50 knees impacted by osteoarthritis, Giorgini et al. concluded that a single injection of autologous mFAT associated with arthroscopy is a safe and effective method to treat knee osteoarthritis [48]. Outcomes were measured 2 years following the procedure using KOOS and IKDC scales, both of which indicated substantial improvements [48]. In the longest-term follow-up analysis of this, Onorato et al. evaluated the effectiveness of mFAT injections for knee osteoarthritis up to 4 years after treatment [49]. The prospective trials featured 46 patients who had diagnostic arthroscopy and single autologous mFAT injection and were monitored at baseline, 6 months, 1 year, and 4 years after surgery [49]. The study reported an increase in the Lysholm knee score and WOMAC score from baseline, as well as a decrease in VAS pain score from baseline at the 4-year follow-up [49]. There were also no major adverse effects reported, and age, body mass index, and the number of stem cells from injected mFAT were not significantly correlated with the results [49]. Patients with synovitis did, however, show a 75% failure rate. Of the patient sample, 32% (15 subjects) were deemed treatment failures due to the necessity for secondary surgery or further injection therapy [49]. This study marks important insight into the potentially high failure rate of mFAT therapies, with a significant proportion later requiring additional, potentially invasive, therapy. There is thus continued need for longitudinal study of mFAT to determine its effectiveness and durability over the long term.

Some studies have focused on specific conditions that may occur concurrently with knee osteoarthritis. In this regard, Malanga et al. conducted a prospective pilot study to determine the safety and efficacy of mFAT for patients with knee pain secondary to osteoarthritis and meniscus tears [50]. Meniscus tears are a common injury that can increase the risk of developing knee osteoarthritis. In the study, twenty eligible patients were assessed at 3, 6, and 12 months following ultrasound-guided intra-meniscal and intra-articular mFAT injections using the Numeric Pain Scale (NPS) and KOOS [50]. Researchers reported a significant improvement in patient pain by one year following the procedure, as well as better KOOSs [50]. While most subjects were not reported to have any major complications, one patient developed uncomplicated cellulitis at the adipose harvest site and was treated with oral antibiotics [50].

Other researchers have also taken a special interest in the elderly population. Gobbi et al. found that a single mFAT injection led to functional, clinical, and quality of life improvements in geriatric patients two years after treatment [51]. There were also no significant complications [51]. Li et al. also found positive effects from mFAT for atherosclerosis and osteoarthritis in the elderly patient population [52].

Nonetheless, there remains a need for further long-term evaluation of mFAT efficacy and potential complications. The majority of studies also feature small sample sizes, therefore pointing to the need for significantly larger randomized controlled trials to truly develop a deep base of information on mFAT safety and effectiveness. Moreover, it is worth noting that while most studies did not report any major complications, the issues that have arisen (e.g., uncomplicated cellulitis at the harvest site) must be further investigated. Advanced imaging techniques such as T2 mapping and T1ρ imaging, as well as the incorporation of quantitative MRI biomarkers, may also prove beneficial in improving understanding of mFAT efficacy.

### 2.3. Hip Osteoarthritis

In addition to knee osteoarthritis, the mFAT biologic has also found use in various other orthopedic applications, with hip osteoarthritis another prominent course of study. Heidari et al. compared the effect of mFAT and a combination therapy of mFAT and PRP for the treatment of hip osteoarthritis [43]. The observational study, which involved 147 patients with hip osteoarthritis, saw significant improvements in both the Oxford Hip Score and visual analog score for pain for both treatment types [43]. Investigators note that the combination therapy may be particularly relevant for patients with low body mass index where it might be difficult to obtain enough mFAT for effective single-biologic dosage [43].

Zaffagnini et al. included 30 patients with hip osteoarthritis in a study featuring single mFAT injection [53]. Clinical evaluations using the VAS, WOMAC, and Harris Hip Score were conducted at baseline and 1, 3, 6, and 12 months after administration [53]. Investigators did not report any major complications, and the majority of patients saw an improvement in WOMAC scores at 1 and 3 months and all other scores at every follow-up compared to baseline [53]. Three patients were noted to have failed, with failure defined as the need for subsequent injection or surgical procedure(s) due to continued or worsened symptoms. Researchers saw better outcomes for mild cases of hip osteoarthritis compared to moderate as assessed via WOMAC scores compared to baseline [53]. Of note, while researchers did not find disease progression in imaging studies, there was also no indication of structural changes to suggest the improvements indicated by the pain scores [53].

Some mid-term characterization of mFAT treatment for hip osteoarthritis has also been conducted. Natali et al. assessed 55 patients with hip osteoarthritis who underwent an ultrasound-guided injection of mFAT. After 3 years, improvements were seen according to the Oxford Hip Score [54]. As in the case of knee osteoarthritis, the studies that exist surrounding the use of mFAT for hip osteoarthritis suffer from a lack of large, randomized controlled trials that are truly capable of deepening the knowledge pool around safety and efficacy for patients over the long term.

### 2.4. Tendon Disease and Ligament Injury

Outside of osteoarthritis as a large category, there has also been limited study of the effect of mFAT as a treatment for tendon disease. In a pilot study, Hogaboom et al. investigated mFAT injections for rotator cuff tendinopathy in 10 spinal cord-injured wheelchair user patients [55]. After ultrasound-guided injections of mFAT into the rotator cuff tendons and other shoulder structures such as the acromioclavicular and glenohumeral joints and subacromial bursa, patients were followed up with at 6 and 12 months [55]. Using the Numerical Rating Scale (NRS), Wheelchair User’s Shoulder Pain Index (WUSPI), Brief Pain Inventory pain interference items (BPI-I7), Patient Global Impression of Change (PGIC), and ultrasound, investigators found meaningful improvements as well as no major adverse effects [55].

Ferracini et al. also conducted a case–control study on Achilles tendon repair when treated with mFAT [56]. Eight patients underwent open suture repair alongside perilesional administration of mFAT. Compared to a group that only underwent the conventional open suture intervention, no differences were seen between the mFAT and non-mFAT groups according to the American Orthopedic Foot and Ankle Society (AOFAS) score and Foot and Ankle Disability Index (FADI) [56]. However, ultrasound evaluation at 3 months showed enhanced tendon remodeling in the group with the mFAT combination therapy [56]. There were also no adverse effects reported for either patient group [56]. Notably, then, while the limited data seems promising regarding the use of mFAT biologic interventions for tendon disease, there must be larger and randomized controlled trials with longer follow-up times to fully characterize the effectiveness and safety profile.

While the majority of studies on the usage and efficacy of mFAT have centered around osteoarthritis symptom alleviation and progression delay, some researchers have also begun expanding in scope to review its potential for other injury types. In a randomized controlled trial that is still ongoing, Wang et al. studied 70 patients with acute anterior cruciate ligament (ACL) injuries [57]. Common among sports-related injuries, ACL issues often require surgical reconstruction to regain the necessary joint function and stability, but the inflammation and graft-to-bone healing still pose challenges during the recovery phase. As such, researchers suggest that mFAT has the potential to promote tissue regeneration and reduce inflammation for ACL injuries. Patients in the study are split into a standard ACL reconstruction group and an ACL reconstruction with mFAT intra-articular injection group [57]. Follow-ups for this continuing trial will be conducted at 3, 6, and 12 months following the procedure, assessed via MRI, Visual Analog Scale scores, International Knee Documentation Committee scores, and inflammatory markers [57]. The results for this trial have not yet been published [57].

### 2.5. Summary and Quality of Clinical Evidence

The single placebo-controlled randomized controlled trial performed by Richter et al. marks the strongest available evidence for mFAT efficacy in knee osteoarthritis [45]. The trials conducted by Ulivi et al. and Baria et al. offer helpful contextual information about the relative efficacy of mFAT when compared to other methods such as corticosteroids and PRP, but they do not confirm superiority over a placebo [18,26]. Prospective cohort studies and retrospective studies are susceptible to selection bias, concerns about placebo effect, and regression to the mean, limiting the information that can be gleaned, though these studies can be helpful to gain insight into mFAT safety and real-world response rates. Further, it is important to note that while pilot studies in tendon disease and hip OA are promising and indicate the need for further research, they remain comparably underpowered. The systematic review by Hohmann et al. similarly concluded that while early clinical outcomes appear encouraging, the overall quality of evidence remains limited due to these ongoing concerns [58]. As such, the positive findings reported across mFAT studies ought to be approached with the necessary caution until further work that addresses these design gaps confirms them.

### 2.6. Comparisons to Other Biologics

As one of many autologous biologics with the potential to promote tissue healing and wound regeneration, mFAT has also been compared against other promising biologics. Interestingly, Mautner et al. found that while both mFAT and bone marrow aspirate concentrate (BMAC) injections could relieve knee osteoarthritis, there were no significant differences between the two [59]. In a systematic review and meta-analysis, Park et al. found that across the six randomized controlled trials included, PRP and mFAT both reached the clinically significant threshold in improving outcomes up to one year following treatment, with similar results for pain alleviation, functional improvement, and safety [60]. At 6 months, however, mFAT showed improvements over PRP by a small but statistically significant margin [60].

Baria et al. found in a randomized controlled trial of 58 patients with symptomatic knee OA, scoring Kellgren–Lawrence grades I–IV, that there was no significant difference between mFAT and PRP at 6 months, with both resulting in clinically meaningful improvement for patients [61]. Nonetheless, the authors note that there are significant mechanistic differences between PRP and mFAT, with the latter providing a more complex tissue product with structural biologic scaffolding, rather than a pure soluble-factor concentrate [61]. Further, PRP is noted to be simpler, less invasive, and potentially cheaper, introducing an economic consideration for the use of PRP over mFAT if neither is clinically superior [61]. Similar comparisons were made to BMAC.

Nonetheless, the comparisons between mFAT and biologics such as PRP and BMAC require further study to determine which are optimal for specific applications and patient populations and needs, as present studies and reviews have indicated comparable but not necessarily superior effects by mFAT over other biologics [58,62].

Autologous chondrocyte implantation (ACI) is a two-stage surgical procedure used to treat full-thickness cartilage defects in the knee, involving the harvesting, culturing, and re-implantation of healthy cartilage cells from the patient [63]. While ACI features significantly more research and longitudinal understanding about its benefits in improving knee osteoarthritis over the long term, it is more invasive than mFAT and involves a surgical procedure [64]. Direct clinical comparisons between the two are still limited and require further study.

### 2.7. Current Regulations

While mFAT is not yet approved by the Food and Drug Administration (FDA) in the United States, the mechanical and not enzymatic nature of processing to produce mFAT renders parts of the process in line with the FDA’s guidelines for “minimal manipulation” of the sample and “homologous use” [50,65]. Homologous use is defined by the FDA as utilization of a tissue for the same basic function in the recipient as in the donor [65]. Since adipose tissue is considered by the FDA to primarily provide cushioning and structural support, it is that certain applications of mFAT may not qualify under these guidelines [65].

Internationally, different regulatory groups monitor mFAT production and use with significant variation. For example, within the European Union, adipose-derived biologics may fall under Advanced Therapy Medicinal Product (ATMP) regulations monitored by the European Medicines Agency (EMA), depending on the degree of manipulation and intended use [66,67]. In the United Kingdom, oversight is provided by the Medicines and Healthcare products Regulatory Agency (MHRA) [68].

As such, there still remain concerns regarding regulation and compliance in commercial use of mFAT outside of research purposes, but the simpler preparation process may be a positive indicator.

**Table 3:** Summary of clinical studies evaluating mFAT in orthopedic applications.

Study	Design	Key Findings
Knee Osteoarthritis		
De Groote et al., 2025 [14]	Longitudinal (n = 39), single mFAT injection	Improved all KOOS domains after 12 mo, peak at 6 mo, with sex differences observed; 18% transient synovitis
Hudetz et al., 2017 [41]	Prospective non-randomized (n = 17, 32 knees), mFAT injection	Increased GAG content in cartilage after 12 mo
Stanciu et al., 2025 [44]	Retrospective observational (n = 335), single mFAT injection	Sustained improvements by 3 years; limited by attrition
Van Genechten et al., 2021 [15]	Prospective study (n = 64), single mFAT injection	TRR 64% (3 mo), 45% (12 mo); moderate durability; 79% transient inflammation
Richter et al., 2025 [45]	RCT (n = 75), mFAT injection	Significant pain/function improvement vs control by 12 mo; longer effect than steroids
Ulivi et al., 2023 [18]	RCT (n = 78), mFAT and arthroscopy	Improved functional and imaging outcomes by 24 mo
Cattaneo et al., 2018 [47]	Prospective (n = 38), mFAT and arthroscopy	Consistent functional improvement across 12 mo
Giorgini et al., 2022 [48]	Retrospective (n = 49), mFAT and arthroscopy	Sustained improvement across 2 years
Onorato et al., 2024 [49]	Prospective (n = 46), mFAT and arthroscopy	Long-term improvement across 4 years; 32% failure rate
Malanga et al., 2020 [50]	Pilot (n = 20), intra-articular and intra-meniscal mFAT	Significant pain and function improvement by 12 mo
Mautner et al., 2019 [59]	Comparative cohort study (n = 111 knees), mFAT vs. BMAC	Comparable efficacy to BMAC
***Hip Osteoarthritis***		
Heidari et al., 2021 [43]	Observational (n = 147), mFAT with and without PRP	Both groups improved up to 2 years; combo may help low BMI patients
Zaffagnini et al., 2025 [53]	Prospective pilot (n = 30), single mFAT injection	Symptom improvement by 12 mo; better in mild OA; no structural MRI changes
Natali et al., 2022 [54]	Observational (n = 55), mFAT injection	Sustained improvement assessed by the Oxford Hip Score for 3 years
***Tendon Disorders***		
Hogaboom et al., 2021 [55]	Pilot (n = 10), mFAT injection into rotator cuff	Pain and functional improvement after 12 mo
Ferracini et al., 2022 [56]	Case–control (n = 8), mFAT and surgical repair	No functional difference with or without mFAT; improved tendon remodeling after 3 mo
***Other***		
Wang et al., 2025 [57]	RCT protocol (n = 70), ACL reconstruction and mFAT	Study ongoing with results pending

## 3. Discussion

### Limitations and Future Directions

Though mFAT could present promising avenues for future exploration, it remains crucial to recognize the limitations that remain and are reflected in this manuscript. In particular, several limitations should be considered when interpreting the current body of evidence on mFAT. Most clinical studies that exist are small, single-arm, and heterogeneous in osteoarthritis severity, processing system, injected volume, concomitant procedures, and follow-up duration, resulting in an inability to perform robust comparisons and synthesis across the mFAT landscape. Further, only one placebo-controlled randomized trial has been published to date, and there is a strong need for further studies to address this gap.

The lack of consistency across mFAT study also expands to outcomes and risks. Both measures vary widely across studies, and, in the case of adverse event reporting, most investigations are concerned primarily with noting only the absence of major complications rather than systematically characterizing minor or transient events. 

Evidence for hip osteoarthritis and tendon pathology remains particularly limited, and long-term data beyond 2–3 years are scarce, making it difficult to draw true conclusions about the lasting power and safety of mFAT. Further, cellular and cytokine composition can vary meaningfully across commercial processing devices, complicating direct comparisons between studies. 

In light of these limitations in mFAT studies and their reflections in this paper, future research should prioritize large, standardized, placebo-controlled randomized trials. Investigations further ought to be marked by harmonized processing protocols, standardized outcome measure frameworks and adverse event reporting processes, as well as extended follow-up for more years following administration of treatment. To better characterize both safety and efficacy in short- and long-term studies, comparative trials against PRP, BMAC, and other existing treatment protocols, along with mechanistic in vivo studies, will also be important. In these studies, optimal indications, dosing, and durability of effect should also be clarified.

## 4. Conclusions

According to existing preclinical and clinical evidence, mFAT presents a promising biologic therapy for orthopedic regenerative medicine. Related to its rich cellular composition—including various factors necessary to promote tissue healing and reduce inflammation—and intact extracellular matrix, mFAT may have positive future indications for clinical use in knee and hip osteoarthritis, as well as tendon disease and other similar injuries. In promoting faster healing and potentially slowing the progression of certain diseases, mFAT could improve patient quality of life.

Despite its promise, mFAT still requires further study and characterization. In particular, there have been limited studies assessing the long-term efficacy and safety of such treatments, and osteoarthritis remains an incurable condition. Further, the current body of literature is limited by relatively small sample sizes, heterogeneous study designs, and other issues with depth, breadth, and standardization. Indeed, a recent systematic review by Hohmann et al. similarly concluded that while early clinical outcomes appear encouraging, the overall quality of evidence remains limited by these concerns [58].

Interpretation of the current clinical literature is complicated by substantial heterogeneity across published studies. Included investigations differed considerably in patient osteoarthritis severity (including variable Kellgren–Lawrence grading), processing systems used for mFAT preparation, injected volumes, concomitant procedures such as arthroscopy, and duration of follow-up. Outcome measures also varied substantially, with studies utilizing KOOS, WOMAC, VAS, Oxford Knee Score, Lysholm score, IKDC, and other assessment tools. These variations pose significant challenges in developing a systematic understanding.

Additionally, though most studies reported favorable safety profiles with few serious complications, adverse event reporting was inconsistent across the literature. Many investigations reported only on the absence of major complications while providing limited characterization of minor or transient adverse events. Standardized adverse event reporting frameworks will therefore be important in future randomized controlled trials to better characterize the safety profile of mFAT therapies. The economic limitations of mFAT usage must also be considered prior to broader usage.

As such, the therapeutic possibilities of mFAT remain not fully understood. Future investigations should prioritize large, randomized controlled trials with standardized processing, administration, and other protocols, as well as featuring longer follow-up periods. Evaluation of the mechanisms of action and pathways in vivo will also be crucial for better dosing and safety understandings. Moreover, comparative studies evaluating mFAT against other biologic therapies such as PRP and BMAC will be beneficial in creating a base of interconnected understanding across biologics. As regenerative medicine continues to evolve at a rapid pace, mFAT may present itself as a strong, minimally invasive therapeutic option capable of addressing many musculoskeletal degeneration concerns. With time and further research, mFAT may indicate future use in the clinic and hospital space.

## Data Availability

No new data were created or analyzed in this study. Data sharing is not applicable to this article.

## Abbreviations

ACI	autologous chondrocyte implantation
ACL	anterior cruciate ligament
AOFAS	American Orthopedic Foot and Ankle Society
ASC	adipose-derived stem cells
BMAC	bone marrow aspirate concentrate
BMI	body mass index
BPI-I7	Brief Pain Inventory pain interference items
CCL2	C-C motif chemokine ligand 2
CCL3	C-C motif chemokine ligand 3
dGEMRIC	delayed gadolinium-enhanced magnetic resonance imaging of cartilage
ECM	extracellular matrix
FADI	Foot and Ankle Disability Index
FDA	Food and Drug Administration
GAG	glycosaminoglycan
IgG	immunoglobulin G
IKDC	International Knee Documentation Committee
KL	Kellgren–Lawrence
KOOS	Knee injury and Osteoarthritis Outcome Score
KSS	Knee Society Score
mFAT	micro-fragmented adipose tissue
MRI	magnetic resonance imaging
MSCs	mesenchymal stem cells
NSAIDs	non-steroidal anti-inflammatory drugs
NPS	Numeric Pain Scale
NRS	Numerical Rating Scale
OKS	Oxford Knee Score
PBS	phosphate-buffered saline
PGIC	Patient Global Impression of Change
PRP	platelet-rich plasma
SVF	stromal vascular fraction
TRR	therapeutic response rate
VAS	Visual Analog Scale
WOMAC	Western Ontario and McMaster Universities Osteoarthritis Index
WUSPI	Wheelchair User’s Shoulder Pain Index
